# Development of Hollow Fiber Membranes Functionalized with Ionic Liquids for Enhanced CO_2_ Separation

**DOI:** 10.1021/acssuschemeng.4c04597

**Published:** 2024-07-31

**Authors:** Julia A. Piotrowska, Christian Jordan, Michael Harasek, Katharina Bica-Schröder

**Affiliations:** † Institute of Applied Synthetic Chemistry, TU Wien, Getreidemarkt 9/163, Vienna 1060, Austria; ‡ Institute of Chemical, Environmental and Bioscience Engineering, TU Wien, Getreidemarkt 9/E166, Vienna 1060, Austria

**Keywords:** carbon capture, ionic liquids, hollow fibers, gas separation, membrane coating

## Abstract

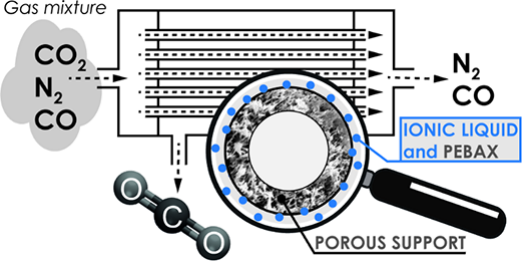

The combination of CO_2_-selective ionic liquids (ILs) with block copolymers, such as Pebax 1657, has demonstrated an enhancement of the gas separation capabilities of polymeric membranes. In the current work, the development of composite membranes by applying a thin, concentrated selective layer made of Pebax/imidazolium-based ionic liquids (ILs) is presented. The objective of the experiments was to determine the optimized IL loading and investigate how the alteration of the anion impacts the properties of the membranes. Two membrane configurations have been studied: coated flat sheet membranes, supported on a porous poly(ether sulfone) (PES) layer, as well as composite hollow fiber membranes, supported on commercial polypropylene (PP) hollow fibers. Coated hollow fiber composites were fabricated using a continuous coating method, offering a straightforward scalability in the manufacturing process. The determined mechanical pressure stability of hollow fiber composites reached up to 5 bar, indicating their potential for various industrial gas separation applications. It was found that the Pebax 1657-based coating containing 40 wt % [C_6_mim][NTf_2_] yielded membranes with the best gas separation properties, for both the coated flat sheet and the hollow fiber configurations. The CO_2_ permeance of hollow fibers reached 23.29 GPU, whereas the CO_2_/N_2_ ideal selectivity stood at 8.7, suggesting the necessity of the further enhancement of the coating technique, which can be achieved, for example, through application of multiple coatings. Nonetheless, the superior ideal selectivity of the CO_2_/CO separation, reaching 12.44, gave a promising outlook for further novel membrane applications, which involve the separation of the aforementioned gases.

## Abstract 2

This article presents the novel approach toward the fabrication of carbon dioxide-selective coated hollow fiber membranes, which can be applied for the sustainable membrane-based CO_2_ separation.

## Introduction

Anthropogenic emissions of carbon dioxide (CO_2_) are the dominant causative agent of global warming. In order to attenuate its detrimental consequences, several strategies have been developed: application of renewable energies, switching to fuels with lower carbon intensity, and an implementation of CO_2_ capture and storage/utilization (CCS/CCU) technologies.^1^ In recent years, various CCS systems have been investigated.^2^ Among numerous CO_2_ separation methods such as adsorption, physical absorption on porous materials, amine-based chemical absorption, or cryogenic distillation, membrane-based technologies have attracted considerable attention.^3^ Membrane-based gas separation exhibits remarkable advantages, such as simple operation and maintenance, no solvent exploitation, reduced capital and operational costs,^4,5^ compact design, and modularity, resulting in the straightforward scalability.^6−8^ Additionally, since in membrane-based processes, no phase change is required, they are typically considered as energy-efficient.^9^ Membranes are utilized in various applications, including air separation, natural gas sweetening, or hydrogen production.^10^ Since CO, CO_2_, and N_2_ are the byproducts of blast furnace gas, their separation becomes crucial.^11^ Moreover, CO separation is particularly prominent as it is a primary output of electrocatalytic CO_2_ reduction for H_2_ production, a key technology for achieving carbon neutrality.^12^ Despite their multiple advantages, membrane-based technologies still demand further enhancement to become fully competitive with the state-of-the-art separation strategy: amine adsorption. Their economic viability relies on membrane selectivity. Membranes are preferred when the high purity of gas streams is not crucial. Their most significant disadvantages are low permeability, selectivity and their trade-off relationship, poor thermal stability, and reduced resistance against harsh, corrosive chemicals. Moreover, they might undergo plasticization, which leads to their deteriorated performance over time.^7,13,14^

The evaluation of membrane performance is based on two crucial parameters: selectivity and permeability. Selectivity determines the separation efficiency, while permeability is decisive for the productivity of a membrane.^15^ The mechanism of CO_2_ transport through polymeric membranes has been already intensively studied and well-described.^16^

Recently, a multitude of membrane modification strategies have emerged, including the formation of polymer blends^17,18^ and mixed matrix membranes.^19,20^ Membrane enhancement can be achieved through the incorporation of metal–organic frameworks,^21,22^ zeolite imidazolate frameworks,^23,24^ addition of polyethylene glycol (PEG),^25^ or graphene oxide in conjunction with carbon nanotubes.^26,27^ Another highly promising concept involves the integration of room-temperature ionic liquids (ILs) into membrane technology. The key factor of ILs is their remarkable selectivity for CO_2_ uptake and separation, attributed to the enhanced solubility of CO_2_ in ILs. It originates from the combination of asymmetrical anions and cations, which create the physio- or chemisorption pathways.^15,28^ Moreover, ILs exhibit low vapor pressure, minimal volatility, high thermal stability, and low flammability.^29^

Among the plethora of ILs, imidazolium-, ammonium-, pyridine-, and phosphorus-based ILs demonstrate beneficial traits for CO_2_ separation.^28,30^ Imidazolium-based cations give rise to high diffusivities and permeabilities of CO_2_, whereas anions impact the CO_2_ solubility^15^ and the viscosity of ILs strongly. Enhanced gas diffusion is expected in less viscous systems due to the lower mass transfer resistance based on higher gas diffusion coefficients. Therefore, the interplay between the cation and anion determines the ultimate CO_2_ separation efficiency, offering significant tunability in the design of IL-based membranes. Among the extensively researched and favored ILs, [C_6_mim][NTf_2_] stands out, with multiple studies confirming its exceptional capacity for CO_2_ solution.^31−33^ Recently, various configuration concepts combining membranes with ionic liquids (ILs) have emerged. These include the use of pure ionic liquids, the creation of dense, polymerized IL membranes, and the development of supported IL membranes.^30^ The direct deposition of ILs on an inert porous substrate results in the formation of supported ionic liquid membranes. Such membranes exhibit high permeability and selectivity toward CO_2_. They are also very attractive due to their easy preparation and handling. However, supported ILs suffer from the high risk of IL leaching, especially when the membrane operates at an elevated temperature or pressure. One of the most promising solutions is the formation of polymer-IL composites in which ionic liquids are immobilized in the polymeric matrix. It eliminates the risk of IL leaching from the porous support; thus, the polymer/IL composite may still operate safely at higher pressure differences.^33^ For the immobilization of ILs, different polymers can be applied. One notably well-studied polymeric material in this context is Pebax, which is renowned for its excellent CO_2_ separation characteristics. Pebax is a trade name for poly(ether-*block*-amide), a member of the thermoplastic elastomer family. It consists of soft, polar polyether–poly(ethylene oxide) (PEO) segments together with rigid, aliphatic polyamide (PA) segments. The PEO part is responsible for the high CO_2_ solubility and its easy permeation, whereas hard PA segments provide high mechanical stability. Transport of gas molecules takes place mainly through PEO parts.^34−36^

Several investigations of the gas transport properties of Pebax/IL freestanding flat membranes have been conducted.^36−38^ These studies have demonstrated enhanced gas transport properties achieved through the incorporation of ILs. Nonetheless, IL-based freestanding membranes suffer from low mechanical stability, which makes them unsuitable for industrial applications. To address this challenge, a potential solution involves the application of IL/Pebax blends onto a porous, highly permeable substrate.^24,39^ Within these configurations, a thin selective layer of IL/Pebax is formed on top of a highly porous, usually polymeric substrate. These structures, known also as composite membranes, offer a potential solution to overcome the constraints associated with the permeance/selectivity trade-off, along with the issue of low mechanical stability. Nevertheless, the penetration of coating material into the porous structure and the potential existence of defects in a thin selective IL/Pebax layer exhibited a detrimental impact on the separation process^10^ and need to be solved when manufacturing such membranes.

Thin-layer composite membranes may exist in various configurations. The geometry of the porous membrane support influences the characteristics of the membrane significantly. IL/Pebax-coated flat sheet membranes were recently described in several studies.^40−42^ As an example, Pishva and Hassanajili^39^ investigated the effect of IL addition to dual-layer composite membranes, with PES as a support and [C_2_mim][BF_4_]/Pebax 1657 as a coating, formed by a cocasting method. This approach was employed to explore the separation of CO_2_ from light gases, utilizing the strong affinity of both CO_2_ and the ionic liquid (IL) to Pebax. As an alternative to the dual-layer composites, Pebax-based composite membranes with ILs encapsulated in porous carbon particles were recently proposed by Silva et al.^43^ In such a configuration, the leaching of ionic liquid was inhibited, resulting in outstanding CO_2_ separation properties and high long-term membrane stability.

Flat sheet membranes are favored due to their simple design and easy preparation. Therefore, the experimental screening and basic research focus on this particular configuration of a membrane module. However, flat sheet membranes come with the drawback of a relatively low surface area, rendering them less practical for industrial applications. In contrast, hollow fiber membranes offer an increased surface area per unit volume and higher packing density and are more cost-effective.^44^ To the best of the authors’ knowledge, the use of polymeric hollow fibers coated with Pebax/IL solutions has not been yet widely investigated. Fam et al.^45^ reported the development of Pebax 1657/[C_2_mim][BF_4_] gel membranes in the form of thin-film composite hollow fiber membranes, which exhibited outstanding CO_2_ separation efficiency.

In the current work, a novel continuous coating technique was introduced to form the uniform Pebax/IL coatings on polymeric porous hollow fibers. The overall aim was to merge the advantageous geometry of hollow fibers with improved gas separation properties of the coated membranes. As the prephase studies, the flat sheet membranes, coated with analogous Pebax/IL solutions, were tested due to their easier manufacturing and handling. For the coating material, Pebax 1657-based solutions, enriched with different contents of two imidazolium-based ionic liquids, namely, [C_6_mim][NTf_2_] and [C_6_mim][Cl], were applied. The objective was to investigate how the alteration of the ionic liquid content and the anion type affects the gas separation properties and to determine the favored IL content. Moreover, the morphology and interaction between Pebax and the IL in the coating were investigated. Subsequently, based on the initial study results, the hollow fibers were coated and tested. The ultimate objective was to render the membranes suitable and scalable for the efficient CO_2_ separation from N_2_ and CO.

## Experimental Section

### Materials and Methods

**Scheme 1:**
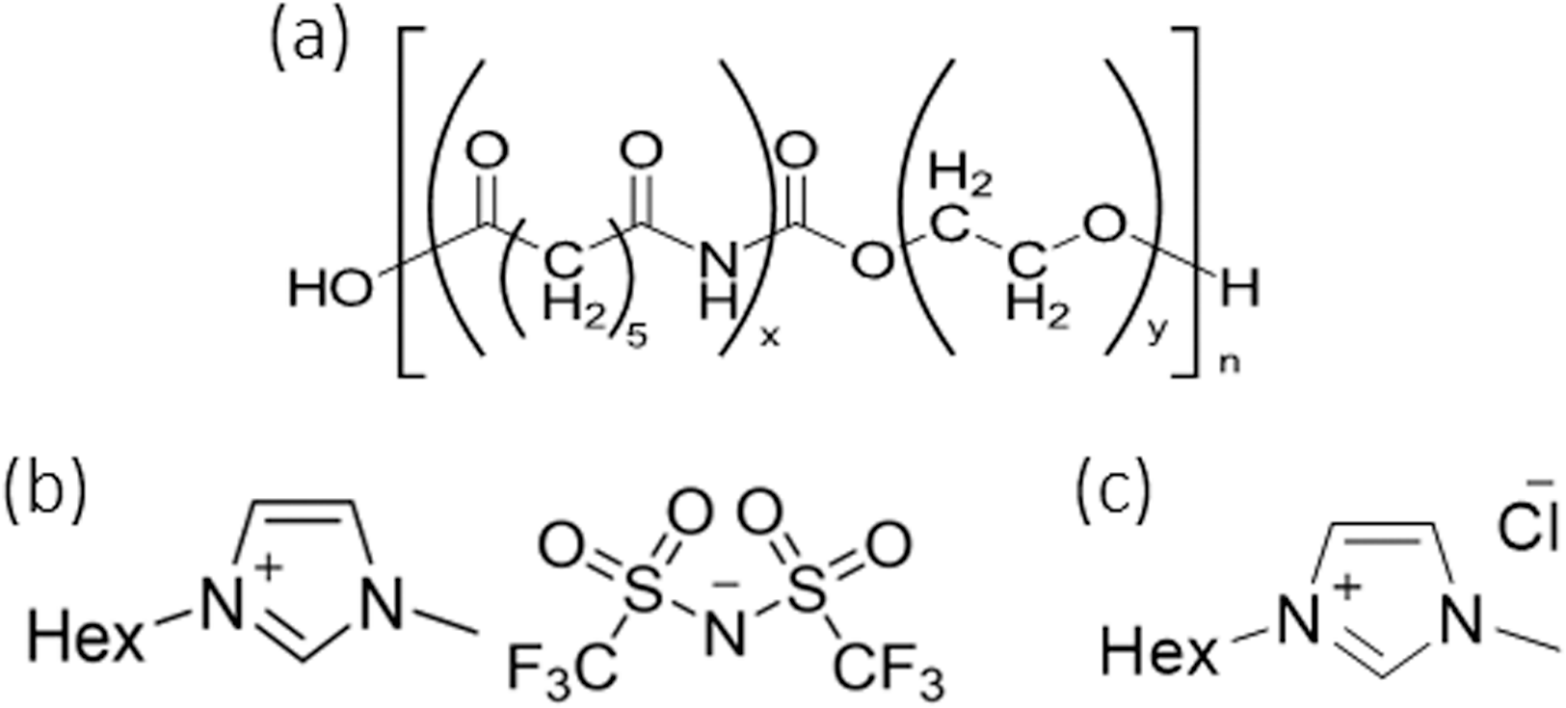
Molecular Structures of Pebax 1657 (a), [C_6_mim][NTf_2_] (b), and [C_6_mim]Cl (c)

For the preparation of the porous supports, pellets of poly(ether sulfone) (PES) of high polymerization grade (7600) were kindly donated by Sumimoto Chemical Europe. As the solvent, *N*-methyl-2-pyrrolidone (NMP) from Bartelt GmbH was used. For the fabrication of a dense coating layer, Pebax MH 1657 was kindly provided by Arkema High-Performance Polymers. Pellets were dissolved in the mixture of deionized water and ethanol (EtOH) with a purity of 99.5 vol % from Merck. The ionic liquids 1-hexyl-3-methylimidazolium bis(trifluoromethylsulfonyl)imide ([C_6_mim][NTf_2_]) with a purity of 99.5 vol % and 1-hexyl-3-methylimidazolium chloride ([C_6_mim][Cl]) with a purity of >98 vol % were purchased from IoLiTec Ionic Liquids Technologies. Viscosity–density data of the ionic liquids, provided by the supplier, are listed in the Supporting Information (see Table S1). Measured FT-IR and ^1^H NMR spectra of both ionic liquids can be found in Figures S1 and S2 in the Supporting Information. Chemical structures of Pebax 1657 and ionic liquids are given in Scheme 1. For gas permeation tests, carbon dioxide and nitrogen with a purity of >99.995 vol % and carbon monoxide with a purity of 99.3 vol % were purchased from Messer Austria GmbH.

### Preparation of Composite Flat Sheet Membranes

The porous PES support was prepared by the phase inversion method. A 20 wt % solution of PES 7600 was obtained by dissolving the predried polymeric pellets in NMP. The mixture was stirred at 60 °C for 24 h. After degassing in an ultrasonic bath, the solution was left to settle for 2 h to ensure the complete removal of air bubbles. Afterward, membranes were cast by pouring the solution on a glass support. The thickness of membranes was controlled with the help of a casting knife film applicator. The gap clearance was adjusted to 100 μm. Subsequently, the plates were immediately immersed in an aqueous coagulation bath and left for 24 h to precipitate completely. Finally, membranes were removed from the glass plate and dried under ambient conditions for 48 h.

For the formation of selective dense layers, a 8 wt % Pebax coating solution was prepared. Predried (at 70 °C, for 24 h) Pebax 1657 pellets were dissolved in the mixture of ethanol and deionized water (of 70/30 wt % ratio). The solution was vigorously stirred at 70 °C for 24 h. Subsequently, the mixture was cooled down to room temperature, and the ionic liquid, namely, [C_6_mim][NTf_2_] or [C_6_mim][Cl], was added and stirred for at least 2 h. The fractions of IL in the solution were equal to 0, 10, 20, 40, 60 and 80% by weight of the Pebax mass. Composite flat sheet membranes were prepared via the dip coating method. PES supports were immersed in the coating solution for 30 s and dried under ambient conditions. Finally, samples were placed in the vacuum oven at 60 °C for 4 h.

### Characterization of Composite Flat Sheet Membranes

The morphology of coated membranes was investigated with the help of scanning electron microscopy (SEM) (EM-30, COXEM). The cross-cut section of membranes was obtained by freezing them cryogenically in liquid nitrogen followed by their subsequent fracturing. Due to the low conductivity of samples, prior to imaging, they were coated with a thin layer of gold, sputtered by an ion sputter coater (SPT-20, COXEM).

The chemical composition of the top surface of the membrane was investigated by elemental mapping using energy-dispersive X-ray spectroscopy (EDS). Samples were sputtered with platinum and measured by a field-emission scanning electron microscope (FE-SEM) (FEI Quanta FEG 250), equipped with an EDS detector (EDAX-AMETEK Octane Elite 55).

The top surface of membranes was investigated by a Fourier transform infrared spectrometer (FT-IR) (VERTEX 70, Bruker Optics), using an attenuated total reflection method (ATR) at ambient, for a wavelength range of 4000–1000 cm^–1^. Thermal properties of membranes were investigated by thermogravimetric analysis and dynamic scanning calorimetry (TGA-DSC) (Netzsch STA 449 F1 system). The temperature increased from −5 to 550 °C with a rate of 10 °C/min, in a N_2_-controlled atmosphere.

The crystallinity of the top surface of membranes was analyzed at ambient by X-ray diffraction (XRD) (X’Pert MPDII) in Bragg–Brentano geometry using an X’Celerator linear detector and Cu Kα_1,2_ radiation for 2θ from 4 to 45° with a 0.020° step size.

### Determination of Gas Transport Properties of Composite Flat Sheet Membranes

Gas transport properties of membranes were measured in a single-gas permeation test. For each coating solution, at least three membranes were measured. Samples were cut to a round shape to fit a circular module with the diameter of 7.4 cm and an effective surface area of 16.97 cm^2^ and transferred to the stainless-steel housing, containing one inlet and one outlet. Membranes were tested at 25 °C in a dead-end configuration. Permeability and ideal selectivity of the membranes were determined using a constant-volume–variable-pressure method.^46^ Samples were fed with a gas at a pressure of 8 bar. After closing the valve at the ambient permeate side, the time-dependent change of pressure was monitored. To calculate the pure single-gas permeability *P_i_* [Barrer], the following equation (eq 1) was used:
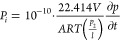
1where *V* stands for the volume of the module at the permeate site [cm^3^], *A* is the effective membrane surface area [cm^2^], 22.414 [cm^3^/mol] is the STP volume per mole of gas, *R* stands for the universal gas constant (6236.56 [cm^3^·cm Hg/mol K]), *T* is the absolute temperature [K], *l* is the average membrane thickness [cm], *p*_2_ is the upstream pressure, and *∂p*/*∂t* is the time differential of pressure build-up on the permeate site [cm·Hg/s]. Membranes were tested with nitrogen (N_2_) and carbon dioxide (CO_2_). The ideal selectivity α [−] for the pair of gases *i* and *j* was calculated from eq 2:
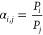
2

### Fabrication and Characterization of IL Composite Hollow Fiber Membranes

Composite hollow fibers were produced via a continuous coating method, using a coating plant, fabricated in-house by TU Wien with financial and consulting support of Axiom, as part of the Austrian FFG funded project “Underground Sun Storage”. As the porous support, commercial polypropylene hollow fibers (with a nominal inner diameter of 0.3 mm and the nominal pore size of 0.4 μm) were used. First, the 15 wt % Pebax coating solutions were prepared by dissolving Pebax pellets in an ethanol/water mixture, as described in the section before. Subsequently, the respective amount of chosen ILs was added to the solution. Based on the results from the initial studies on flat sheet membranes, the chosen compositions of coating solutions contained 0, 10, 20, and 40 wt % [C_6_mim][NTf_2_] or 40 wt % [C_6_mim][Cl], based on the Pebax mass. An exact amount of IL was added to the Pebax solution and stirred, as described previously.

The following conditions of continuous coating were applied: Pebax solution’s temperature of 75 °C, a fiber take-up speed of 20 mm/s, and a 10 mm fiber spacing. During the coating process, a single endless fiber was air-dried, guided through the heated Pebax solution, collected on the rotating hexagonal winder, and left to dry for 48 h at ambient.

The morphology of the single fiber cross section was investigated by SEM. Sample preparation was analogous to that in the case of flat membranes, as described in the previous section.

### Determination of Gas Transport Properties of Hollow Fiber Membranes

Membrane modules were tested in single-gas permeability tests. Forty fibers of 22 cm length were arranged in parallel and rolled into a bundle. Next, they were placed in a stainless-steel housing, and their endings were potted by epoxy glue. For each coating solution, at least three modules were tested. Based on SEM images, the average fiber diameter was estimated, and the total surface area of the membrane was calculated (97.82 cm^2^). For the pure gas permeation tests, the constant-pressure method was applied. Fibers were measured in a dead-end configuration, at 25 °C, for driving pressure differences (Δ*p*) ranging from 2 to 3 bar, with an increment of 0.25 bar. Gas was fed from the shell, and the permeate was collected on the lumen side. The flow rate of gas permeating through the membrane was recorded with the help of a digital flow meter (Definer 220-L from Bios International Corp.).

Single-gas permeability *P_i_* [Barrer] of the hollow fiber composites was calculated using eq 3:
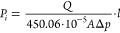
3where *Q* is the volumetric flow rate through the membrane [mL/min], *A* denotes the membrane surface area [cm^2^], Δ*p* is the driving pressure difference [bar], and *l* is the average membrane thickness [μm] of the active layer. Hollow fiber membranes were tested with nitrogen (N_2_), carbon dioxide (CO_2_), and carbon monoxide (CO). Equation 2 was applied to calculate the ideal selectivity α.

## Results and Discussion

### Flat Sheet Membranes

Since the fabrication and characterization of the IL/Pebax selective layers on hollow fiber membranes are of higher complexity, for the initial determination of IL/Pebax coating properties, flat sheet membranes were cast, coated, and characterized according to the methodology described above.

### Morphology and Chemical Composition of Flat Sheet Membranes

**Figure 1:**
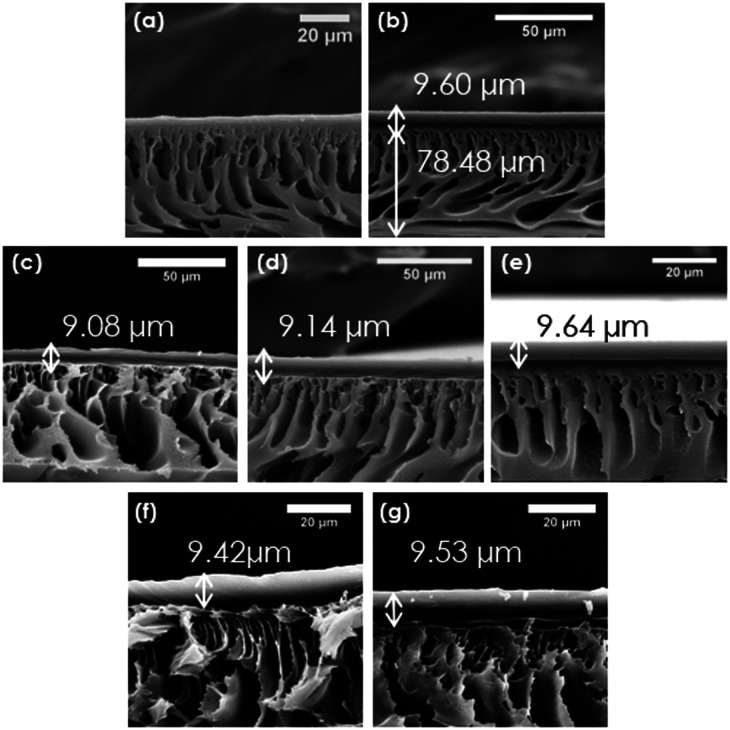
SEM images of the uncoated flat sheet membrane (a), a membrane coated with neat Pebax (b), and membranes coated with 10 (c), 20 (d), 40 (e), 60 (f), and 80 wt % (g) [C_6_mim][NTf_2_] in Pebax 1657.

The morphology of fabricated flat sheet membranes was investigated by SEM. As visible in Figure 1a, which shows the cross section of the uncoated PES substrate, no distinct thin, selective coating layer was observed on the top of the porous support. Thus, it was assumed that transport of gases through the membrane should be nonaffected by the selective coating. The support exhibited finger-like porosity, formed during phase inversion. The measured thickness of the PES support was 78.5 μm. The appearance of the coating layer was observed for the sample coated with the neat Pebax (Figure 1b). Samples coated with Pebax/[C_6_mim][NTF_2_] solutions (Figure 1c–g) also exhibited the presence of a thin, selective layer. In some cases, small areas of delamination between the porous support and a thin layer of coating were visible. Most likely, it occurred during sample preparation, for example, during the fracturing in liquid N_2_. The dense IL/Pebax layer was homogeneous and free from macrodefects, which indicated the uniform distribution of IL in the coating solution. The thickness of the coating ranged from 9.1 ± 0.8 to 9.6 ± 0.3 μm, as given in Table 1.

**Table 1:** Coating Thickness (with Standard Deviations, SD) of Flat Sheet Membranes Coated with Different Contents of [C_6_mim][NTf_2_] and [C_6_mim][Cl]

**IL content[wt %]**	**0**	**10**	**20**	**40**	**60**	**80**
Pebax/[C_6_mim][NTf_2_] thickness ± SD [μm]	9.6 ± 0.3	9.1 ± 0.8	9.1 ± 0.5	9.6 ± 0.5	9.4 ± 0.4	9.5 ± 0.4
Pebax/[C_6_mim][Cl] thickness ± SD [μm]	9.6 ± 0.3	13.7 ± 0.9	15.8 ± 1.5	19.1 ± 1.1	19.0 ± 1.1	20.0 ± 1.2

The thickness of coating was negligibly affected by the addition of [C_6_mim][NTf_2_]. On the contrary, for the samples coated with analogous contents of [C_6_mim][Cl], the coating thickness was significantly influenced by the amount of IL added (see Table 1 and Figure S3 in the Supporting Information). It was attributed to the high viscosity of [C_6_mim][Cl] (3302 cP) and, thus, the overall viscosity of the coating solution. According to the literature,^47−49^ for the higher viscosities of Pebax-based solutions, the formation of thicker coating layers is expected. In general, the [C_6_mim][Cl]-coated layers were of higher thickness than for [C_6_mim][NTf_2_], ranging from 13.7 ± 0.9 to 20.0 ± 1.2 μm. Moreover, considerable deviations of coating thicknesses were also observed. The homogeneity of the selective layer thickness was dependent upon the kind of IL used. To confirm the uniformity of the coating thickness, it was measured at 10 different spots. The average standard deviations (SD) for samples coated with [C_6_mim][NTf_2_] and [C_6_mim][Cl] were 0.5 and 1.2 μm, respectively (for SD values, see Table 1). Although these values were in the acceptable range, they could also suggest the small, possible phase separation and nonperfectly uniform distribution of [C_6_mim][Cl], caused by the presumable accumulation of IL in the swollen PEO domains.^50^

To prove it via visualization of the distribution of IL in Pebax, SEM-EDS analysis of the top surface of samples was carried out for the membranes coated with 80 wt % IL/Pebax blends. Elemental mapping of F and Cl was carried out in order to determine how [C_6_mim][NTf_2_] and [C_6_mim][Cl] respectively were located in the top layer of the coating.

**Figure 2:**
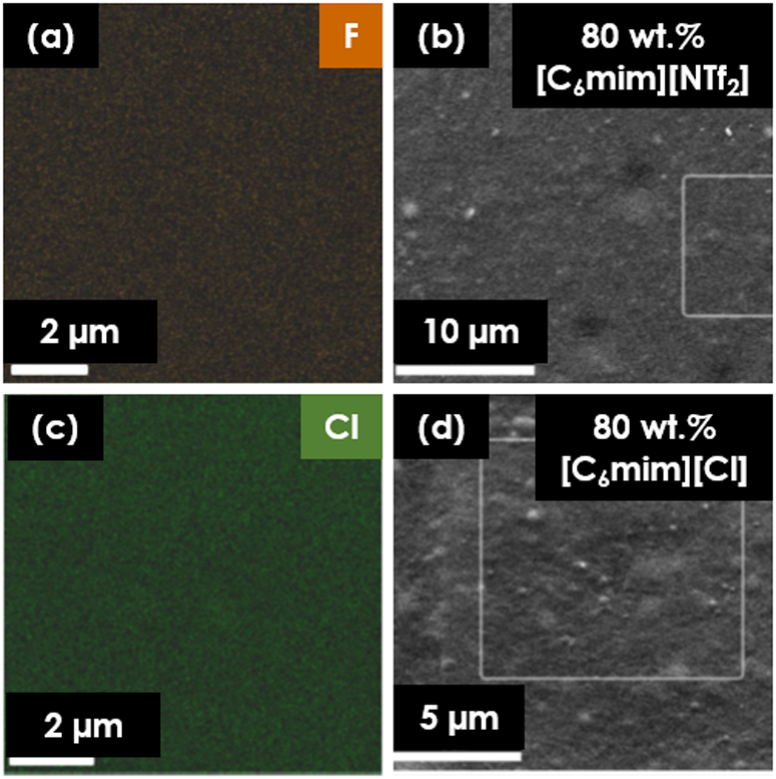
Elemental mapping of fluorine (F) in samples coated with 80 wt % [C_6_mim][NTf_2_]/Pebax (a), chlorine (Cl) mapping of the sample coated with 80 wt % [C_6_mim][Cl]/Pebax (b), and SEM images of top views of coated membranes (c,d).

As visible in Figure 2, no significant formation of concentrated regions of ionic liquids or microseparation was observed. Both [C_6_mim][NTf_2_] and [C_6_mim][Cl] appeared evenly distributed in the coating. Since no phase separation was present for the highest ionic liquid content, it is reasonable to extrapolate that for lower contents of the IL, this phenomenon would not occur as well.

### FT-IR

**Figure 3:**
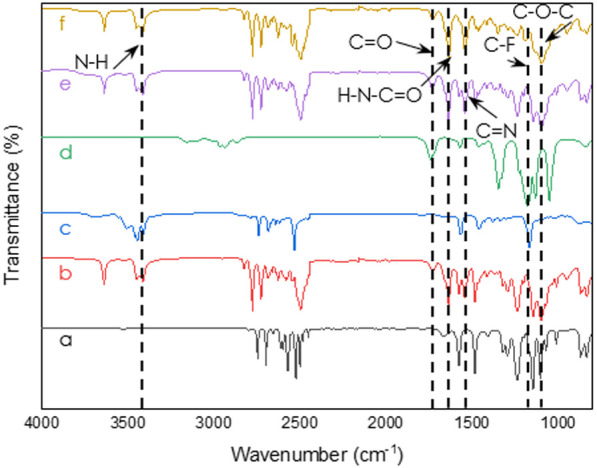
FT-IR spectra of the PES uncoated membrane (a), PES membranes coated with neat solutions of Pebax 1657 (b), [C_6_mim]Cl (c), and [C_6_mim][NTf_2_] (d), and membranes coated with Pebax and 20 wt % IL: [C_6_mim][NTf_2_] (e) and [C_6_mim][Cl] (f).

Figure 3 presents the comparison between the FT-IR results for PES membranes with and without a Pebax coating. FT-IR analysis successfully verified the presence of a Pebax 1657 coating on the PES substrate. This confirmation was supported by the identification of distinctive Pebax characteristic bands in the spectrum, specifically at 3638, 1736, and 1640 cm^–1^. These bands were attributed to the N–H, C=O, and H–N–C=O groups, respectively. The aforementioned groups were located in the PA segment. Additionally, a peak at 1120 cm^–1^ was observed, which was attributed to the vibration of ether groups (C–O–C) in the PEO segment. Furthermore, the FT-IR analysis gave insight into the interaction between Pebax 1657 and ILs. To confirm the presence of ILs’ characteristic bands in Pebax coating, the spectra of neat ionic liquids and samples coated with 20 wt % IL in Pebax were compared. The characteristic bands associated with the ILs used in the experiments were visible in the Pebax/IL spectra. Specifically, a C–F stretch was identified at 1180 cm^–1^, and a C=N stretch was observed at 1565 cm^–1^.^38,39^

### Thermal Properties

The thermal properties of the coated membranes were investigated by two techniques: thermogravimetric analysis (TGA) and differential scanning calorimetry (DSC). The TGA results, presented in Supporting Information, Figure S4, indicated that the decomposition of Pebax occurred at 365 °C. [C_6_mim][Cl] decomposed at 268 °C, while [C_6_mim][NTf_2_] was more thermally stable, reaching the significantly higher decomposition temperature of 435 °C. These results were in good agreement with the literature data.^51,52^

DSC thermograms indicated that melting of pure Pebax 1657 occurred at 206 °C, as can be deduced from the presence of a distinct peak. It was attributed to melting of PA crystallites. On the contrary, the PES support remained stable throughout the entire temperature range under investigation. The glass transition temperature of both PA and PEO segments could not be detected by DSC. Moreover, DSC curves of Pebax/IL-coated membranes did not reveal any distinct phase transition within the temperature range of 0 to 400 °C, indicating the amorphous nature of ILs.

### XRD

To determine the impact of ILs’ addition on the crystallinity of the coating, XRD was measured. XRD patterns of the membrane top surfaces (see Supporting Information, Figure S5) have proven the amorphous nature of the Pebax coating. However, the sharp and distinct signals, at a 2θ of 25.96°, were attributed to the crystalline phase within the Pebax 1657 structure (PA 6 blocks). The addition of both IL types did not result in a significant shift of the peak position. However, for 40 wt % [C_6_mim][NTf_2_], the decline of the signal at 25.96° was observed. Incrementation of the IL content caused a stronger intensity drop, indicating a further decrease of the crystalline phase. The crystallinity drop was justified by the amorphous nature of [C_6_mim][NTf_2_]^53^ (confirmed also by TGA). As a certain amount of IL was added, the volume fraction of Pebax (containing PA crystallites) in the coating decreased. The same but less pronounced tendency was observed for [C_6_mim][Cl].

### Gas Transport Properties of Flat Sheet Membranes

**Figure 4:**
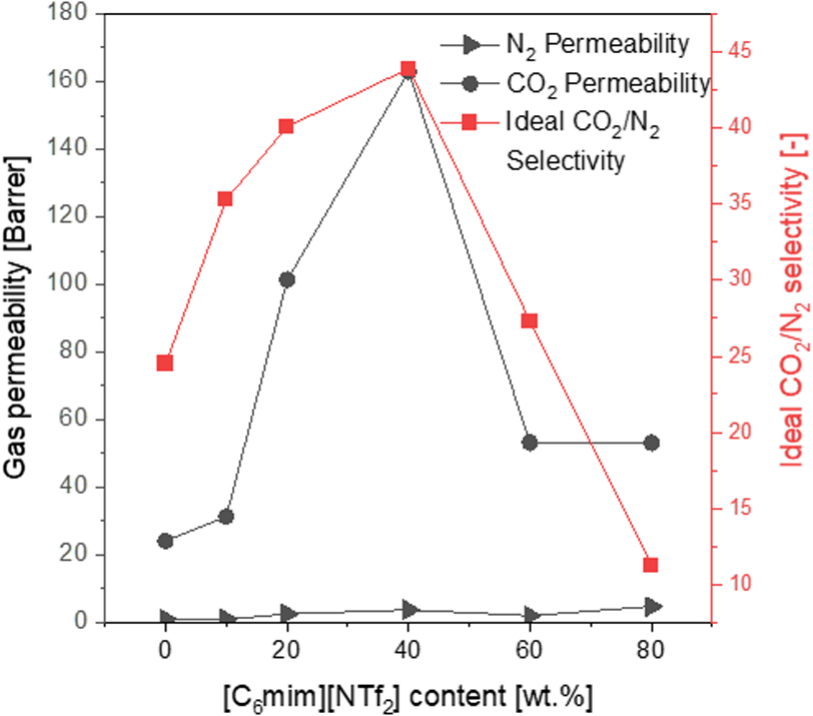
CO_2_ and N_2_ permeabilities (left axis) and CO_2_/N_2_ ideal selectivities (right axis) of flat sheet membranes, as the function of the [C_6_mim][NTf_2_] content.

Prior to coating the fibers, it was essential to determine which IL content would favor CO_2_ separation the most. For that, the gas transport properties of [C_6_mim][NTf_2_]-coated flat sheet membranes were evaluated during single-gas permeation tests. The ideal gas selectivities and permeabilities, depicted in Figure 4, were strongly influenced by the addition of [C_6_mim][NTf_2_]. Initially, the increased gas permeability for a higher IL content was observed. The strong CO_2_ permeance increase was justified by the interactions of polar CO_2_ molecules with the [NTf_2_]^−^ anion, as well as with PEO (Pebax building block), with the dominating IL effect contribution. The permeability of nonpolar N_2_ increased insignificantly, which resulted in the improved CO_2_/N_2_ ideal selectivity.^39^ Nevertheless, this tendency held true until the IL content reached 40 wt %, beyond which a decline in both CO_2_ permeability and ideal selectivity was noted. Although the elevated gas permeabilities for higher IL amounts are typically observed,^38,39^ the drop of CO_2_ permeability after exceeding a certain IL content has already been reported.^39,45^ This phenomenon was explained with the pronounced migration of ionic liquid to the sublayer and resulting pore blockage. A 40 wt % content of [C_6_mim][NTf_2_] yielded the sample of the most optimal properties, with CO_2_ permeability reaching 162.9 Barrer and a CO_2_/N_2_ ideal selectivity of 44. When compared to the literature data for a neat Pebax 1657 film^54^ (see Table S2), where a CO_2_ permeability and CO_2_/N_2_ ideal selectivity of 80 Barrer and 70, respectively, were reported, the presented here 40 wt % [C_6_mim][NTf_2_]/Pebax-coated membranes were more CO_2_-permeable but less CO_2_/N_2_-selective.

### Coated Hollow Fiber Membranes

The overall goal of the research was the fabrication of the CO_2_-selective composite hollow fibers, uniformly coated with the mixture of ionic liquids and Pebax. In the laboratory scale, such membranes are commonly produced by dip coating.^55^ It is a very simple but time-consuming method that does not allow us to obtain membrane modules with large amounts of homogeneously, uniformly coated fibers. Here, a method of continuous hollow fiber coating, in which the single fiber is passed through a heated coating solution and subsequently collected on the hexagonal, rotating winder, allowed us to obtain the homogeneous and defect-free coating layer on the fiber.

**Figure 5:**
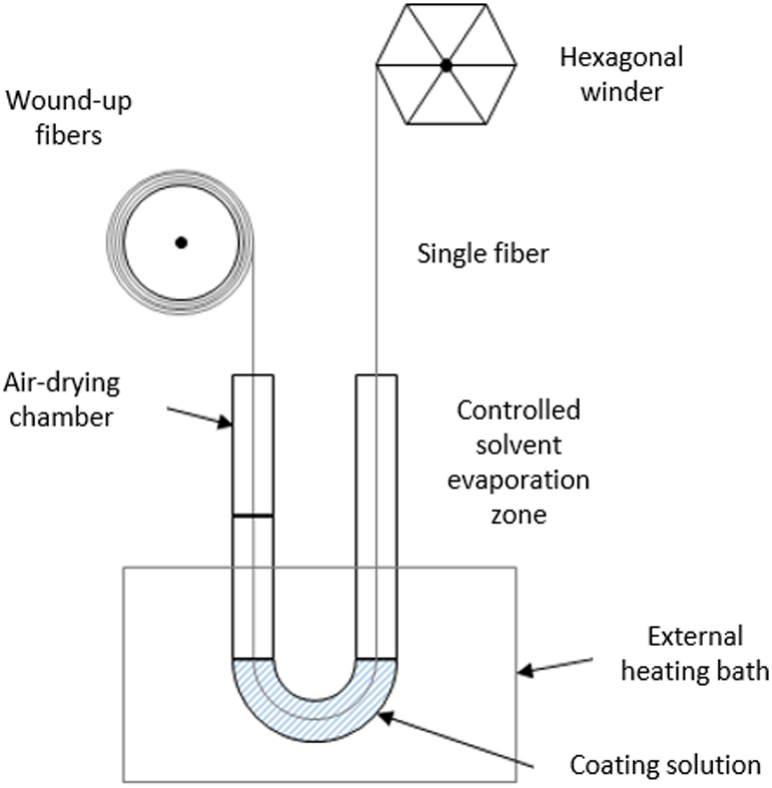
Schematic diagram of the continuous coating process of hollow fibers.

The schematic representation of the coating plant in the laboratories of TU Wien is given in Figure 5.

**Figure 6:**
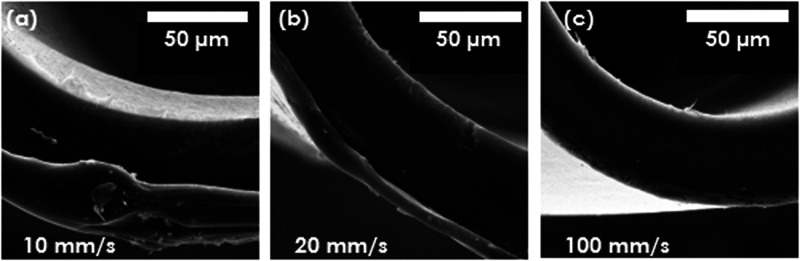
SEM images for different fiber take-up speeds: 10 (a), 20 (b), and 100 mm/s (c).

At the initial stages of the research, it was essential to determine the optimal parameters of the coating procedure. For the porous support, polypropylene (PP) hollow fibers were selected, as they exhibited sufficient mechanical stability and chemical inertness. Commercially available PES fibers turned out to be too fragile, and fibers were disrupted during the coating attempts. It was observed that the fiber collection rate significantly influenced the coating thickness and homogeneity. The impact of fiber take-up speed was clearly visible on SEM images, presented in Figure 6. The higher take-up speed resulted in the unacceptably low coating thickness, whereas when the fiber collection rate was too slow, the formation of large polymer drops on the surface of fibers was observed. This could be explained by the fact that for higher take-up speeds, the residence time of a fiber in coating solution is lower. The collection rate of 20 mm/s resulted in the desired, clot-free, approximately 10 μm-thick coating. Another pivotal parameter was the temperature of the external heating bath. It impacted the viscosity of a coating solution and thus the resultant thickness of the dense layer. Too high viscosity may cause a clogging of tubing, whereas too low viscosity induced poor adhesion between the solution and fiber, resulting in a very low coating thickness. For the chosen Pebax 1657 concentration of 15 wt %, the optimal temperature was 75 °C.

Based on the results for the flat sheet membranes, the investigated scope of IL contents was narrowed down to 0–40 wt %. After the completion of all primary studies, the sequence of coating experiments with various IL contents and two IL types was carried out.

### Morphology of Coated Membranes

**Figure 7:**
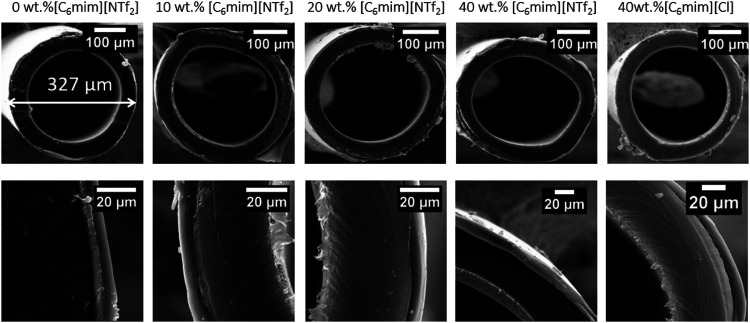
SEM images of cross sections of fibers coated with 0–40 wt % [C_6_mim][NTf_2_] and 40 wt % [C_6_mim][Cl], with magnifications of 300× (top) and 2000× (bottom).

SEM images of fiber cross sections are listed in Figure 7. The average coating thickness values with standard deviations (SD) are given in Table 2. The content of the ionic liquid had no significant impact on the thickness of the coating. Nonetheless, it is worth mentioning that fibers coated with the Pebax/[C_6_mim][Cl] solution exhibited the highest coating thickness, which was also observed for the flat sheet membranes. The thickness of the dense layer for all coating types was approximately 10 μm for all of the samples.

**Table 2:** Average Coating Thickness Values (with Standard Deviations) of Fibers Coated with Different Contents of [C_6_mim][NTf_2_] and [C_6_mim][Cl]

	**[C**_**6**_**mim][NTf**_**2**_**]**				**[C**_**6**_**mim][Cl]**
**IL content[wt %]**	**0**	**10**	**20**	**40**	**40**
coating thickness ± SD [μm]	9.0 ± 1.4	10.2 ± 2.0	8.0 ± 1.9	8.9 ± 2.1	10.5 ± 2.3

### Mechanical Stability of the Membranes

Prior to gas permeation tests of the Pebax/IL-coated samples, the burst test of fibers coated with a pure Pebax solution was carried out. The goal was to determine the maximal pressure difference (Δ*p*) under which fibers can operate without deterioration of their properties. N_2_ was fed with Δ*p* increasing from 2 to 10 bar in a stepwise manner, and the gas flow rate through the membrane was monitored. Fibers were tested in a shell-to-lumen configuration: N_2_ was fed through the outer surface of fiber, and the permeate was collected from the inner side of the fiber.

**Figure 8:**
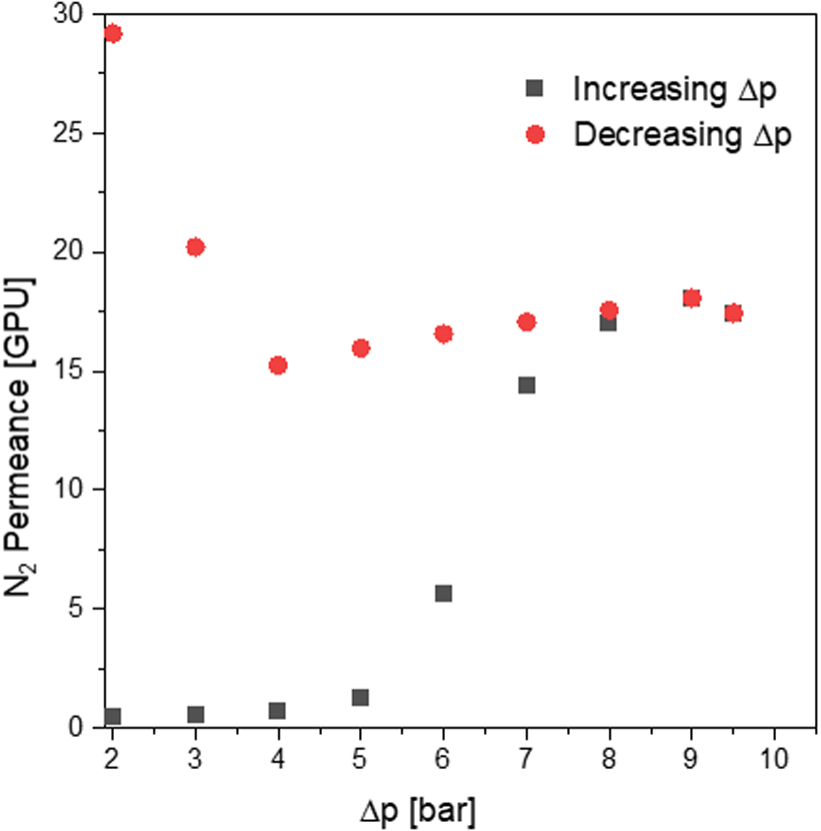
N_2_ permance of the Pebax-coated membrane, measured for Δ*p* ranging from 2 to 10 bar.

**Figure 9:**
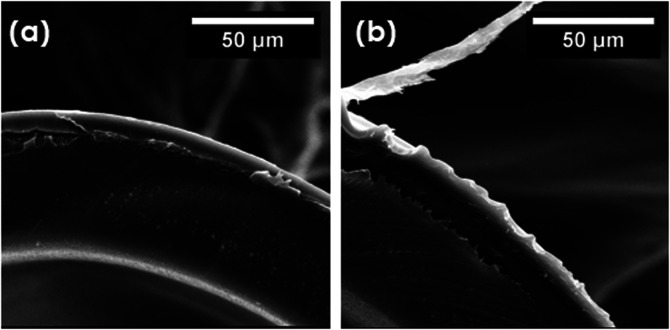
Comparison between the fiber/coating adhesion before (a) and after the test (b).

As visible in Figure 8, after exceeding the Δ*p* of 6 bar, the N_2_ permeance through the membrane increased significantly, which was in contradiction with the prior, linear correlation of Δ*p* and measured permeance. Moreover, the values of flow rates recorded again, at lower Δ*p* values, did not match the previous results. SEM images, presented in Figure 9, indicated the presence of delamination between the layer of coating and the fiber structure. Hence, it was determined that the safe range of the operation pressure difference reached up to 5 bar. Based on that, the safe, specific gas permeation test conditions were established: Δ*p* ranging from 2 to 3 bar with increments of 0.25 bar.

### Effect of Operating Gas Pressure

**Figure 10:**
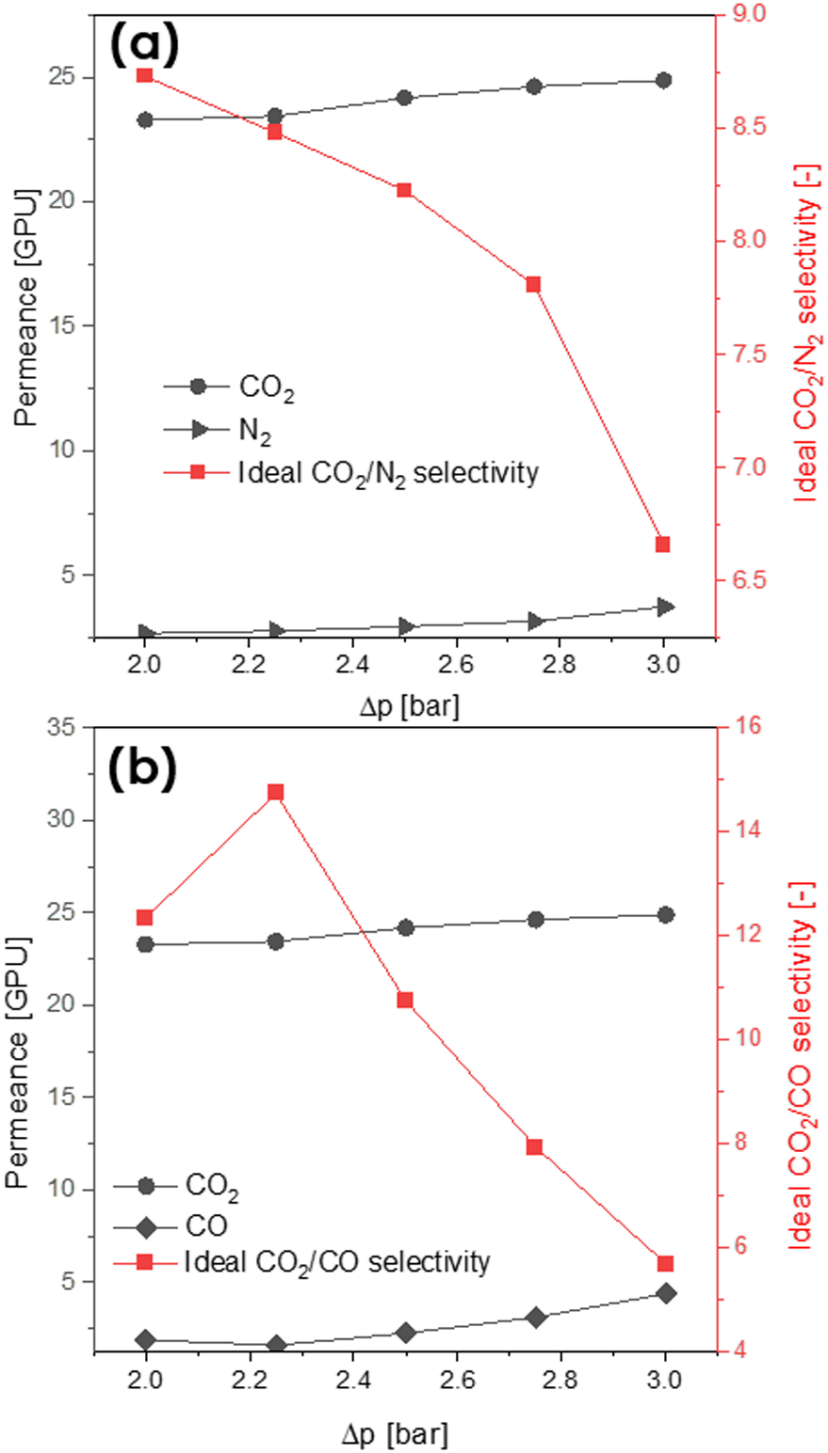
N_2_/CO_2_ (a) and CO/CO_2_ (b) permeances (left axis) and CO_2_/N_2_ (a) and CO_2_/CO (b) ideal selectivities (right axis) of the membrane coated with 40 wt % [C_6_mim][NTf_2_]**/**Pebax solution, measured at different Δ*p* (2–3 bar).

Single-gas permeability tests of IL/Pebax-coated fiber membranes were carried out with N_2_ followed by CO and CO_2_ measurements. For every coating solution, at least three modules, each containing 40 fibers, were tested. As visible in Figure 10, the driving pressure difference influenced both permeabilities and ideal selectivities of the membranes. Flow rates of all gases increased along with the increasing Δ*p* in a linear manner. Moreover, a membrane ideal selectivity drop was observed as Δ*p* increased. This dependency was noticed for each tested sample, for both CO_2_/N_2_ and CO_2_/CO ideal selectivities. Pressure dependence of gas transport through the coated membrane should be mainly driven by the interaction between the polymer and molecules of penetrating gas. Pebax 1657 is a copolymer built from rubbery, soft PEO segments and glassy, stiff PA parts (60/40 wt %, respectively).^36^ Superior gas separation between CO_2_ and light gases like N_2_ of Pebax 1657 can be explained by the high affinity of CO_2_ molecules for PEO segments, specifically for the polar ether linkages.^28,56^ Therefore, it was expected that at higher Δ*p*, CO_2_ should permeate through the membrane with a higher rate, whereas permeability of the nonpolar gases such as CO or N_2_ should remain constant. Here, this tendency was not observed: the permeance of both gases increased when the higher Δ*p* was introduced. Higher permeabilities of samples resulted in their diminished ideal selectivities, which was in accordance with the trade-off selectivity/permeability correlation.^57^ Therefore, the drop of ideal selectivity could be justified by the presumable presence of microdefects in Pebax coating, which expanded once subjected to higher pressures. Hence, the transport mechanism through the membrane was partially governed by the interaction between a highly porous support and gas molecules.

### Effect of the IL Loading

**Figure 11:**
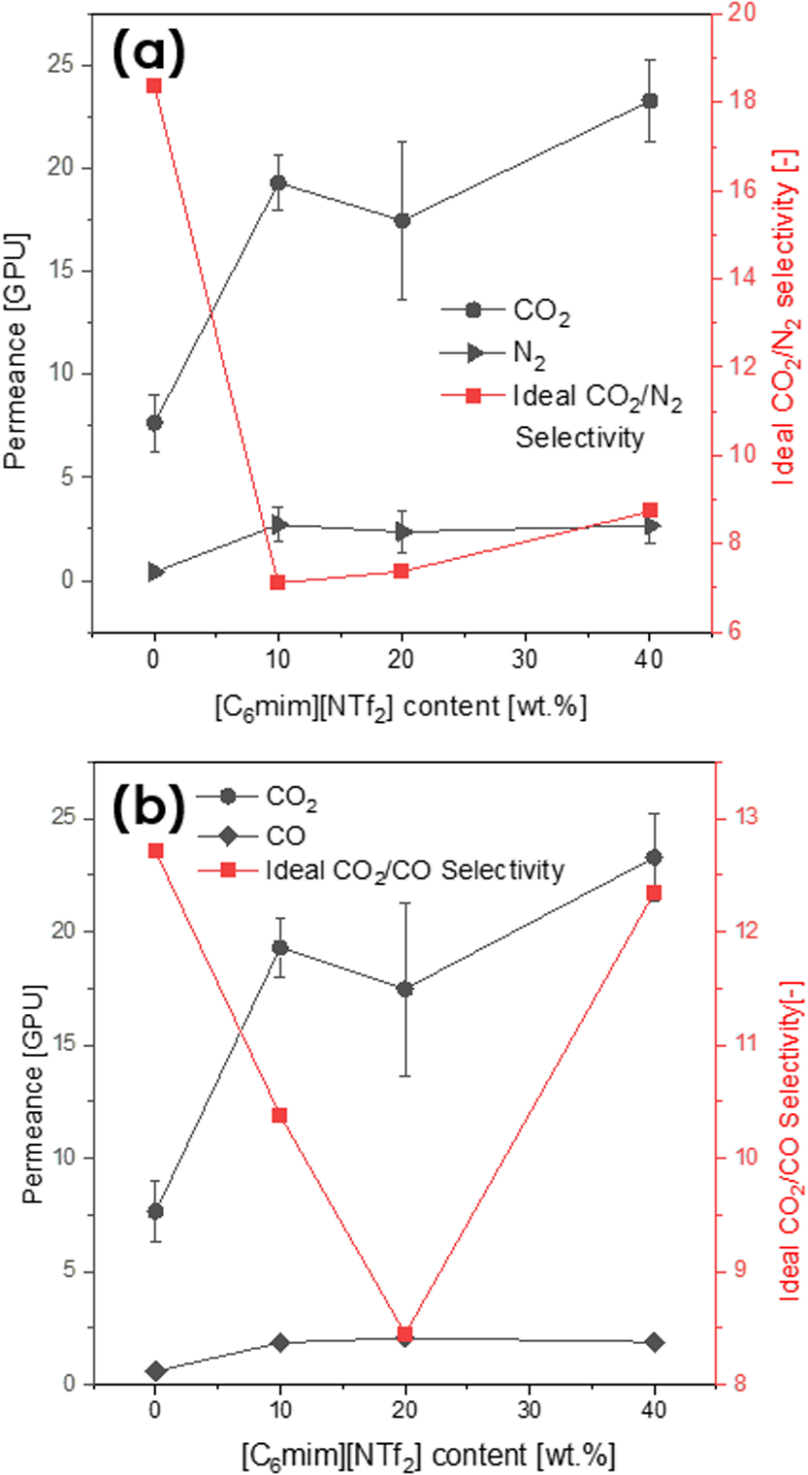
N_2_/CO_2_ (a) and CO/CO_2_ (b) permeances (left axis) and CO_2_/N_2_ (a) and CO_2_/CO (b) ideal selectivities (right axis), measured at a Δ*p* of 2 bar, as the function of the [C_6_mim][NTf_2_] loading.

To determine the impact of the IL content on the transport of gases through the membranes, single-gas permeations of samples coated with 0, 10, 20, and 40 wt % [C_6_mim][NTf_2_] were measured. The exact gas permeances, as well as CO_2_/N_2_ and CO_2_/CO ideal selectivities (measured at a constant Δ*p* of 2 bar), are given in Table 3 and depicted in Figure 11.

**Table 3:** Average Gas Permeances and CO_2_/N_2_ Ideal Selectivities, Measured at a Δ*p* of 2 bar

	**average gas permeance [GPU]**			**average ideal selectivity [−]**	
**[C**_**6**_**mim][NTf**_**2**_**] loading[wt %]**	N_2_	CO_2_	CO	CO_2_/N_2_	CO_2_/CO
0	0.4	7.7	0.6	18.4	12.7
10	2.7	19.3	1.9	7.1	10.4
20	2.4	17.5	2.1	7.4	8.5
40	2.7	23.3	1.9	8.7	12.4

For the neat Pebax coating, the achieved ideal selectivity of 18.4 did not reach the maximum value obtained in the literature. For Pebax-coated fibers, Fam et al.^45^ reported the CO_2_/N_2_ ideal selectivity of 34 (see Supporting Information, Table S2). The diminished membrane performance was potentially caused by the absence of an additional gutter layer. Such a layer could rectify any underlying coating defects, ultimately allowing to attain the higher selectivity.^58^ The formation of multiple coating layers in a continuous process remains an ongoing technical challenge. On the other hand, the ideal selectivity of CO_2_/CO, reaching 12.7, gave a very promising outlook for the novel applications of membranes, which involve the separation of the aforementioned gases. So far, membrane CO_2_/CO separation remains challenging due to the similar solubility and diffusivity of these gases in common polymers.^12^ Unfortunately, there are very few data on membrane CO_2_/CO separation available (see Table S2 in the Supporting Information). For neat Pebax 1657 flat sheets, Park et al.^11^ reported a CO_2_/CO ideal selectivity of 50. Nevertheless, this value cannot be fully compared with the data presented in this work due to the different membrane geometry and type. Missing research on CO separation in hollow fiber membranes highlighted the need to investigate this area further. In this context, the application of Pebax/IL-coated fibers presented here appears as the relevant outlook for further research.

The addition of IL to coating resulted in the considerable rise of gas permeance through the membranes, which was in accordance with the literature.^39^ The 10 wt % [C_6_mim][NTf_2_]/Pebax-coated samples were of higher permeabilities than the ones coated with a neat polymer. This can be explained by the potential increase of the porosity of the dense Pebax layer caused by the introduction of even small IL amounts. Another possible explanation of this phenomenon is the reduction of crystallinity of PEO segments, induced by the addition of ILs, reported in prestudies with flat sheet membranes (see the section XRD) and in the literature.^39,59^ The further incrementation of the IL loading resulted in the higher membrane ideal selectivity. As described in the section “Gas Transport Properties of Flat Sheet Membranes”, this phenomenon was justified by the enhanced interaction between a [NTf_2_]^−^ anion, CO_2_ molecules, and PEO segments, stemming from their high polarity.^59^ Moreover, [C_6_mim][NTf_2_] exhibits the higher CO_2_ solubility than neat Pebax. All of the aforementioned factors contributed to the more pronounced increase of CO_2_ permeance than of N_2_, for which permeances were insignificantly affected by the incremented IL amount. The CO_2_/CO separation in IL/Pebax-coated membranes was mostly supported by the lower solubility of CO in [NTf_2_]^−^.^60^ The CO_2_/CO ideal selectivity of the samples coated with 40 wt % IL was significantly higher than the ones coated with 10–30 wt %.

### Effect of the IL Anion Type

**Figure 12:**
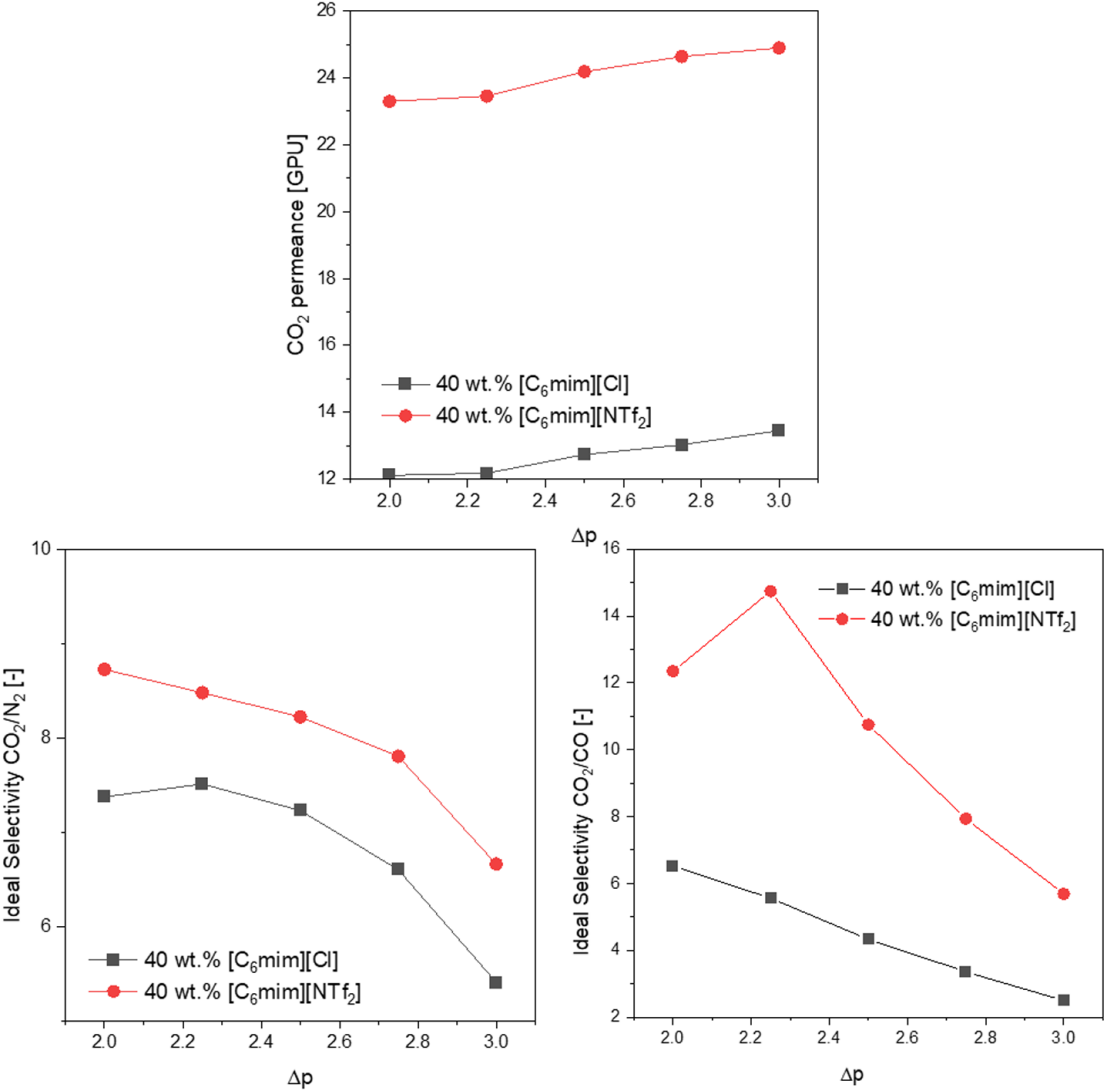
Comparison between the CO_2_ permeance (top) and the CO_2_/N_2_ selectivity of membranes coated with [C_6_mim][NTf_2_] and [C_6_mim][Cl] (bottom).

Hydrophilicity remarkably influences the solubility of CO_2_ in ILs.^61^ Therefore, it was relevant to study how alteration of ILs’ anion into more hydrophilic would influence the properties of the IL/Pebax-coated membranes. The [NTf_2_]^–^ anion gives the rise to the hydrophobic properties of [C_6_mim][NTf_2_], whereas the Cl^–^ anion increases the ILs’ hydrophilicity.^62^ Moreover, the viscosity of [C_6_mim][Cl] (3302 cP) solution is significantly higher than the one of [C_6_mim][NTf_2_] (63.2), which should also contribute to the overall permeance of the coated membranes. After investigating the flat sheet membranes, 40 wt % Pebax mass was determined as the optimal IL loading. The comparison between the 40 wt % [C_6_mim][Cl]/Pebax-coated samples and analogous samples coated using [C_6_mim][Cl]/Pebax solution is depicted in Figure 12. It also shows the impact of the Δ*p*. For both IL types, a linear rise in CO_2_ permeance and a decrease in ideal selectivities were noted as Δ*p* increased. This trend, described in the section “Effect of Operating Gas Pressure”, also held true for [C_6_mim][Cl]. Overall ideal selectivities and CO_2_ permeances were higher for membranes coated with [NTf_2_]-based ILs. Moreover, some of the [C_6_mim][Cl]/Pebax-coated samples exhibited a huge deviation in their quality. This phenomenon, as well as their lower values of ideal selectivity, may be explained by the possible phase separation between Pebax and [C_6_mim][Cl], which led to the nonuniform IL distribution and the reduced homogeneity of the coating layer.

### Long-Term Stability of Fibers

**Figure 13:**
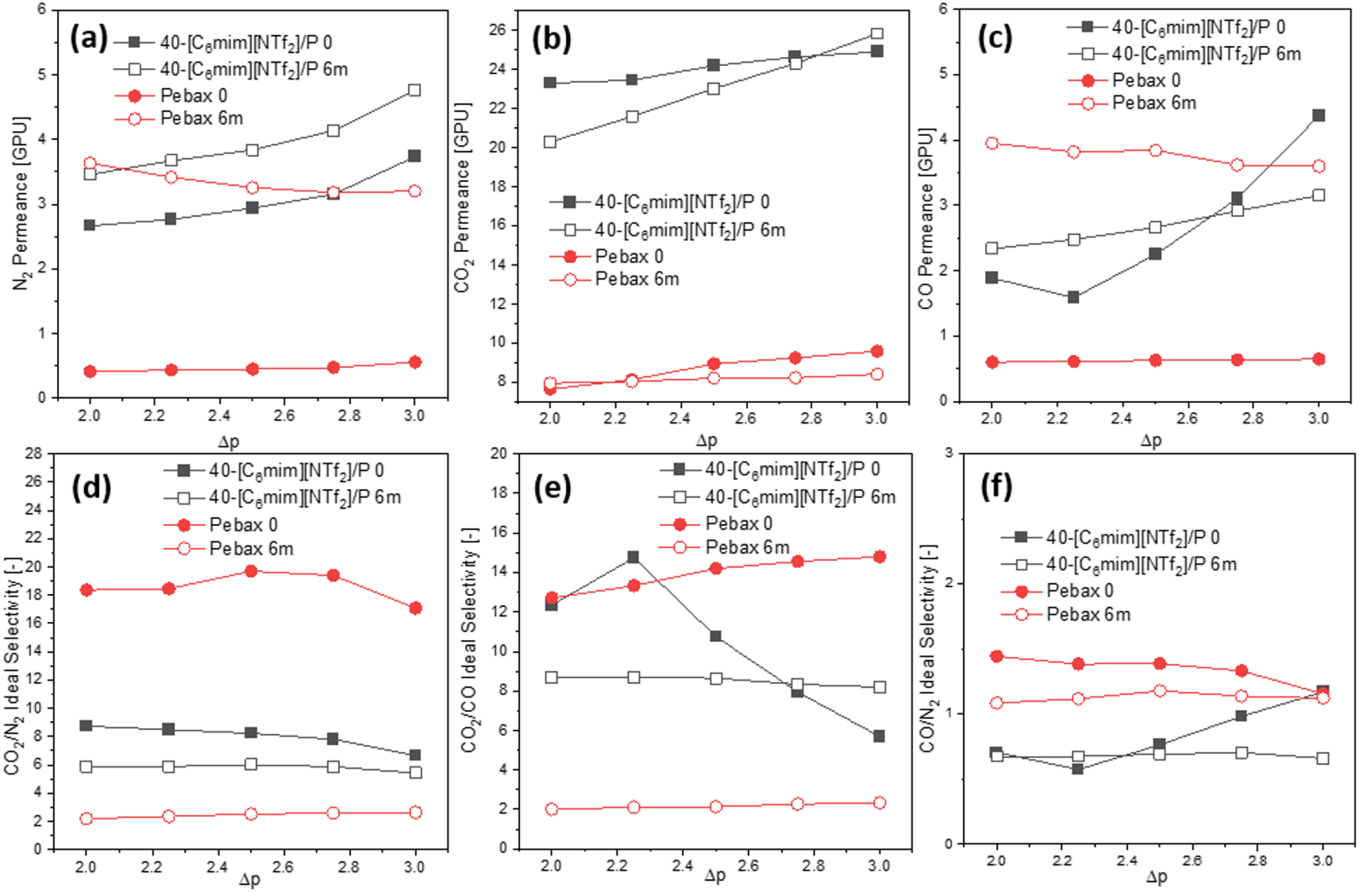
N_2_, CO_2_, and CO permeances (a–c) and ideal selectivities (d–f) of neat Pebax- and 40 wt % [C_6_mim][NTf_2_]/Pebax-coated membranes, measured initially (0) and after 6 months (6m), at varying Δp (2–3 bar).

To address the issue of long-term stability of the membranes, gas permeation experiments tests were repeated for the samples coated with neat Pebax and the 40 wt % IL/Pebax mixture, 6 months after the first measurements. The change of N_2_, CO_2_, and CO permeances and gas pair ideal selectivities is depicted in Figure 13.

For each coating type, after 6 months, higher permeances of all gases were recorded, accompanied by the ideal selectivity drop. Deteriorated performance of samples was most likely caused by plasticization, a phenomenon that occurs when the concentration of CO_2_ in the polymer becomes sufficient to enhance the free volume and mobility of its segments.^63^ After the multiple measurements of samples with CO_2_, sorption of CO_2_ induced the swelling of the membrane, and the overall flow through the membrane became more pronounced: CO_2_, CO, and N_2_ could pass through the membrane easily. Interestingly, it was observed that the addition of IL inhibited the plasticization effect; samples coated with neat Pebax entirely lost their ideal selectivity, whereas for the samples coated with Pebax and IL blends, this drop was less pronounced. Nonetheless, aging of membranes remains an ongoing challenge, which can be addressed in future studies.

### Conclusions and Outlook

In this work, a novel continuous coating method was applied to develop the composite hollow fiber membranes, coated with blends of Pebax 1657 and imidazolium-based ionic liquids. The initial studies on Pebax/IL-coated flat sheet membranes allowed determination of the impact of the addition of ILs to Pebax solution as well as establishment of the most optimal IL content, 40 wt % Pebax mass. For Pebax/IL-coated hollow fibers, the highest ideal selectivities for CO_2_/N_2_ and CO_2_/CO were achieved by the membranes coated with 40 wt % [C_6_mim][NTf_2_], reaching the values of 8.73 and 12.44, respectively. In the context of highly challenging membrane-based separation of CO and CO_2_, the Pebax/IL-coated membranes presented in this paper appear as a very promising solution. Therefore, the further improvement and application of the Pebax/IL-coated hollow fiber membranes for CO separation appear as the relevant outlook of this research. A continuous coating method allowed for the robust fabrication of Pebax/IL-coated hollow fiber membranes, which can be easily upscaled. For the further enhancement of their gas separation properties, multiple coatings, which would heal all of the potential defects of the selective layer, could be introduced. However, the technical aspects of the continuous coating method leave this approach as an open challenge, which authors of this article will address in the nearest future.

Development of efficient and sustainable solutions for CO_2_, CO, and N_2_ separation is essential for both industrial and environmental reasons. The recent research on commercialization of membrane-based systems and their competitiveness with the state-of-the-art technologies reveal their high potential.^7,13,64,65^ Since the membranes presented in this work exhibit promising results, particularly for CO/CO_2_ separation, it would be valuable to analyze their manufacturing costs and sustainability and evaluate their green chemistry metrics. For this, the additional membrane properties, such as mechanical, chemical, thermal, and long-term stability under real operating conditions, together with process design and simulation, are necessary.^65^ The detailed and complete comparison with the existing technologies appears as the relevant and interesting outlook for the work presented in this article.

## Supporting Information Available

Viscosity and density data of ionic liquids; FT-IR and ^1^H NMR spectra for all ionic liquids; SEM images of composite flat sheet membranes; TGA, DSC curves, and XRD spectra of measured composite membranes; comparison of membrane separation properties with literature data (PDF)

## ABBREVIATIONS

CCS: carbon capture and storage
CCU: carbon capture and utilization
PEG: polyethylene glycol
ILs: ionic liquids
[C_6_mim]: 1-hexyl-3-methylimidazolium
[NTf_2_]: bis(trifluoromethylsulfonyl)imide
PEO: poly(ethylene oxide)
PA: polyamide
PES: poly(ether sulfone)
PP: polypropylene
NMP: *N*-methyl-2-pyrrolidone
SEM: scanning electron microscopy
EDS: energy-dispersive X-ray spectroscopy
FT-IR: Fourier transform infrared spectrometry
ATR: attenuated total reflection
TGA: thermogravimetric analysis
DSC: differential scanning calorimetry
HFM: hollow fiber membrane
SD: standard deviation
