# Supplementary material for: Development of Hollow Fiber Membranes Functionalized with Ionic Liquids for Enhanced CO2 Separation
Source: ACS Sustain Chem Eng. 2024 Jul 31;12(32):12236–48. doi: 10.1021/acssuschemeng.4c04597 (PMC11323277; doi:10.1021/acssuschemeng.4c04597)
Supplement: Supplementary file 1 — sc4c04597_si_001.pdf [file sc4c04597_si_001.pdf]

Supporting Information

Development of Hollow-Fiber Membranes Functionalized with Ionic Liquids for Enhanced CO_2 Separation

Julia A. Piotrowska†, Christian Jordan‡, Michael Harasek,‡ and Katharina Schröder*,†*

†Technische Universität Wien, Institute of Applied Synthetic Chemistry, Getreidemarkt 9/163, 1060 Vienna, Austria

‡Technische Universität Wien, Institute of Chemical, Environmental and Bioscience Engineering, Getreidemarkt 9/E166, 1060 Vienna, Austria

Corresponding Authors

* Katharina Schröder, phone: +43 1 58801 163601, mail: katharina.schroeder@tuwien.ac.at

* Michael Harasek, phone: +43 1 58801 - 166 202, mail: michael.harasek@tuwien.ac.at

Number of pages: 7

Number of figures: 5

Number of tables: 2

1. Viscosity-density data, FT-IR and ^{1}H NMR of the pure ionic liquids

Table S1 Viscosity and density data of purchased ionic liquids, provided by the company

IL type / Data type	$[C_6mim][NTf_2]$	$[C_6mim][Cl]$
**Viscosity [cP]**	63.2	3302
**Density [g/cm³]**	1.37	1.04

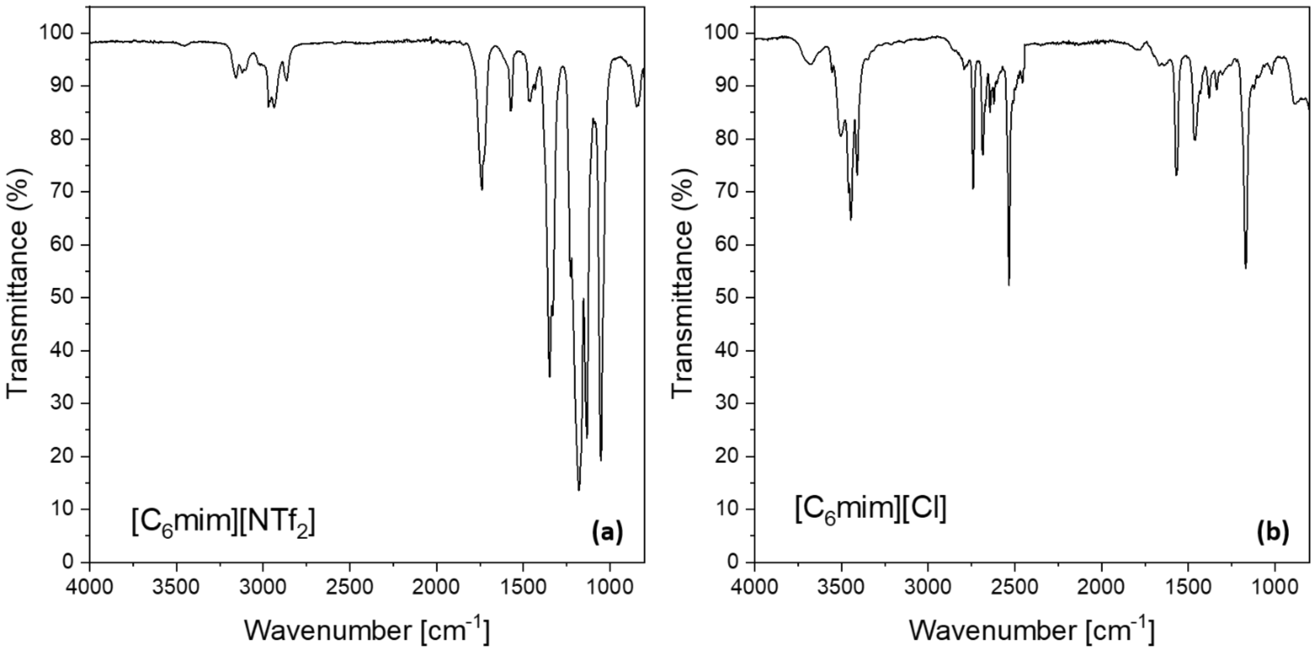

Figure S1. FT-IR spectra of pure $[C_6mim][NTf_2]$ (a) and $[C_6mim][Cl]$ (b)

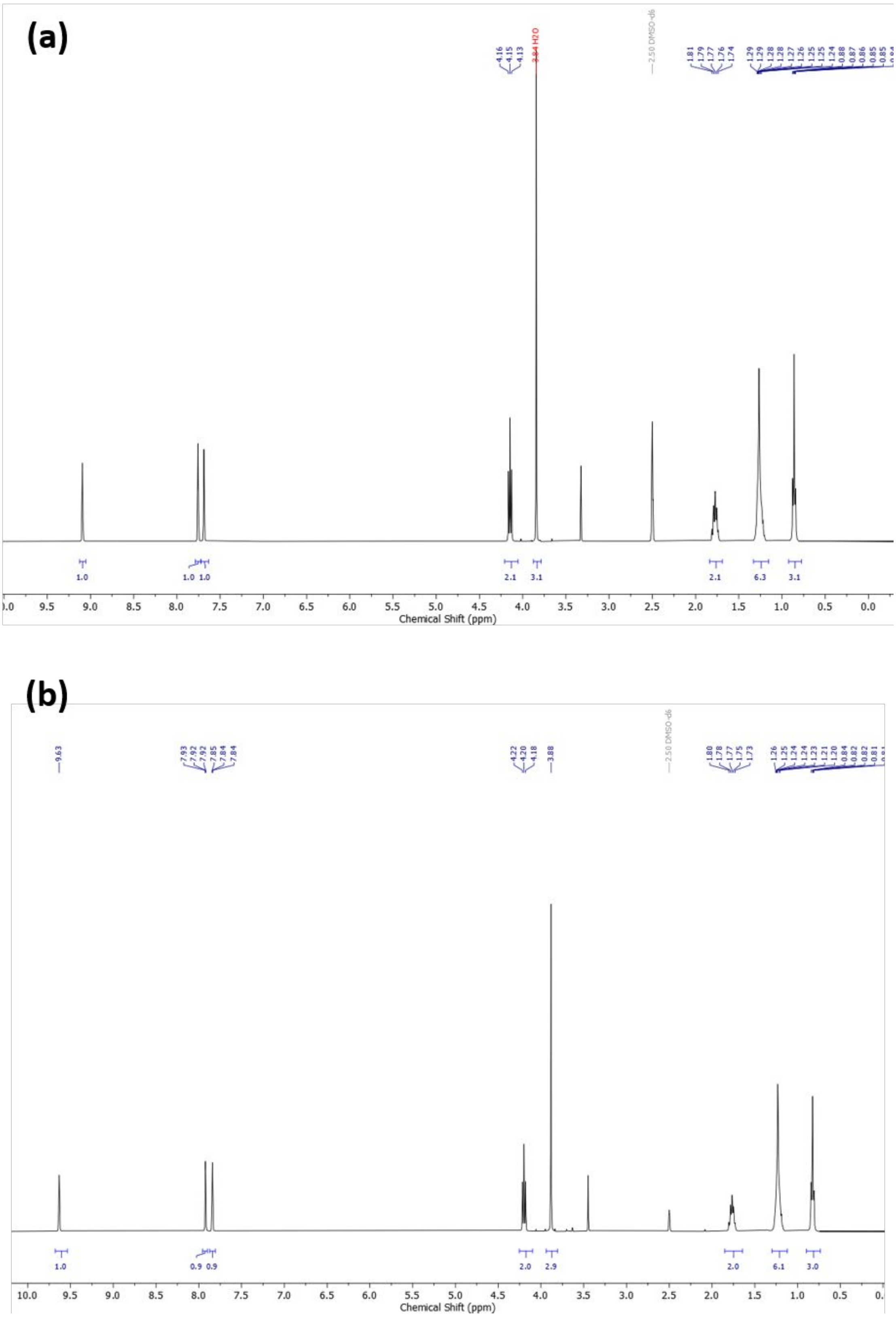

Figure S2. ^{1}H NMR spectra of pure
$[C_6mim][NTf_2]$ (a) and $[C_6mim][Cl]$ (b)

2. SEM images of flat sheet membranes coated with [C_6mim][Cl]

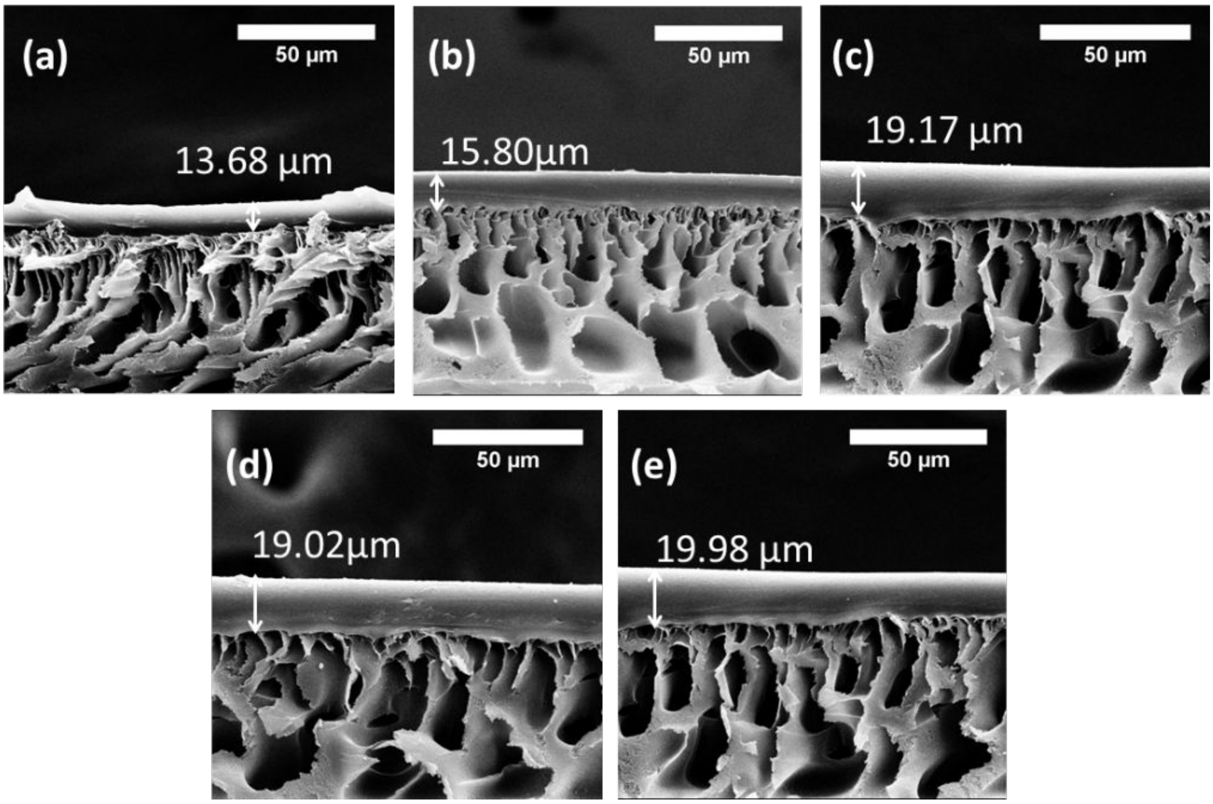

Figure S3. SEM images of flat sheet membranes coated with 10 (a), 20 (b), 40 (c), 60 (d) and 80 wt.% of [C_6mim][Cl] (e)

3. TGA and DSC curves

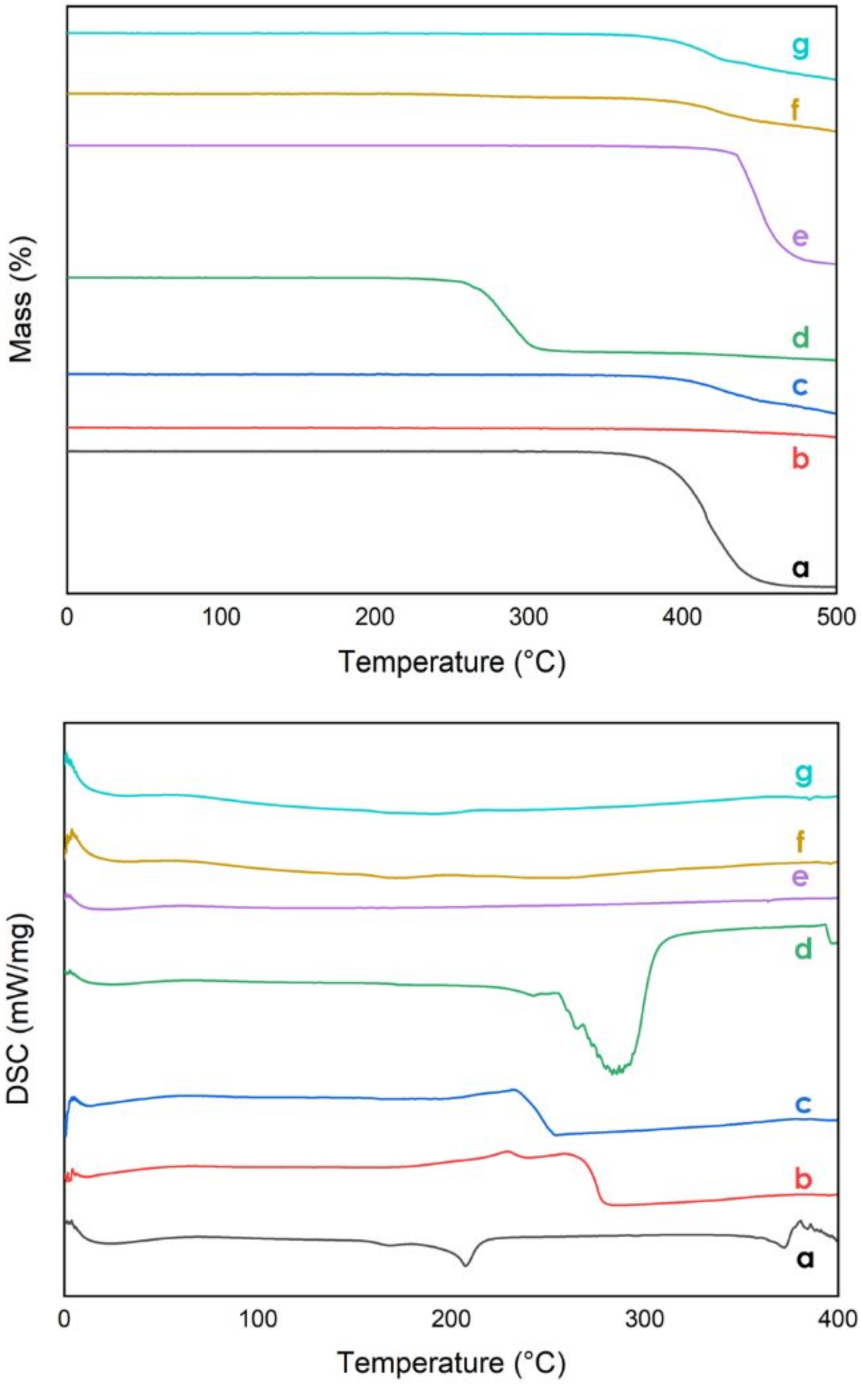

Figure S4. TGA (upper) and DSC (lower) curves of: pure Pebax 1657 (a), PES uncoated membrane (b), membranes coated with: neat Pebax (c), neat $[C_6mim]Cl$ (d), neat $[C_6mim][NTf_2]$ (e), Pebax and 20 wt.% of $[C_6mim][NTf_2]$ (f), Pebax and 20 wt.% of $[C_6mim][Cl]$ (g)

4. XRD spectra of coated membranes

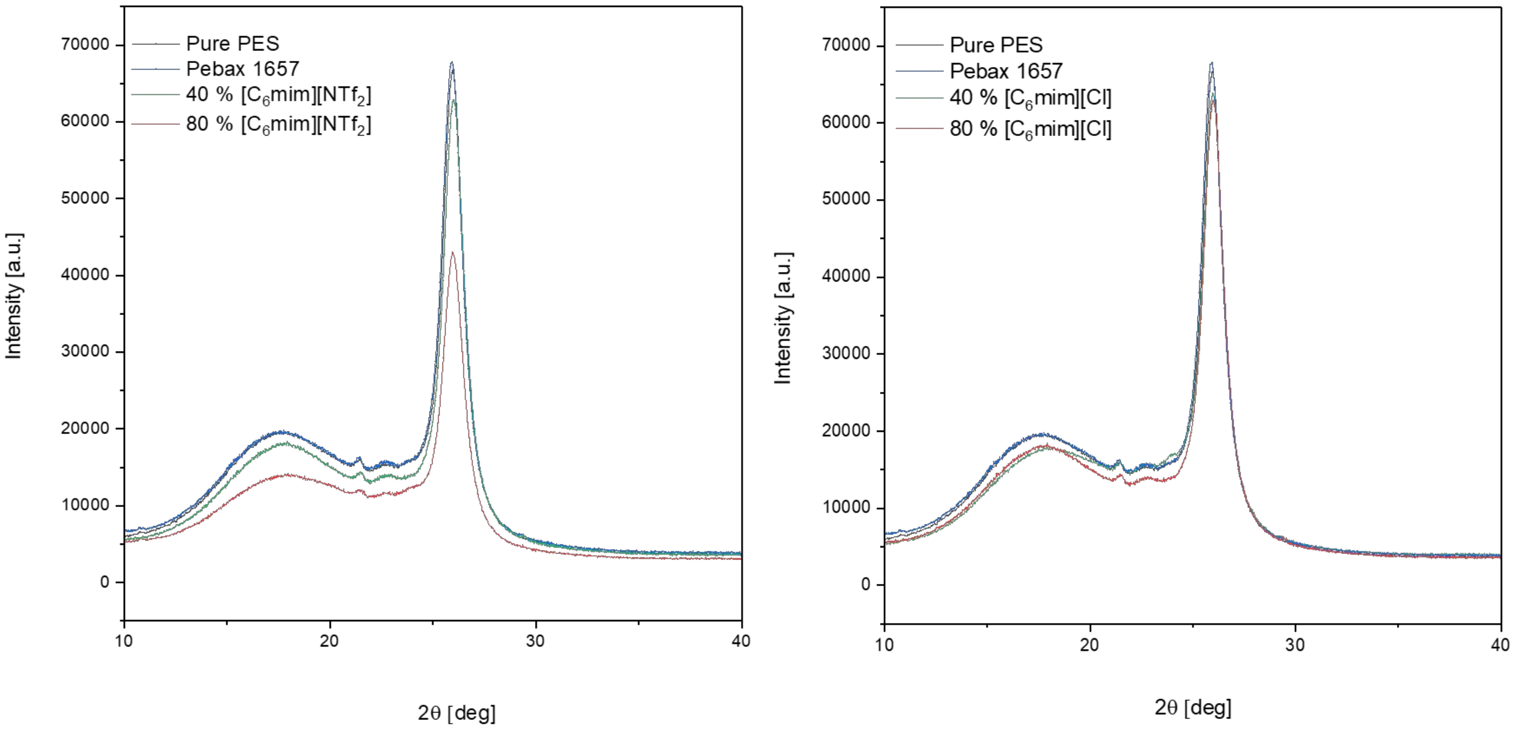

Figure S5. XRD spectra of dense neat Pebax 1657, Pebax1657/IL 40 wt.% and Pebax1657/IL 80 wt.%, for [C_6mim][NTf_2] (a) and [C_6mim][Cl] (b)

5. Comparison of CO_2/N_2- and CO_2/CO-separation properties of membranes with the literature data

Table S2 Literature comparison - performance of membranes coated with Pebax 1657 and / or ILs

	Membrane type	Coating Material	CO_2 permeability	Ideal selectivity [-]			Test conditions	Ref.
				CO_2/N_2	CO_2/CO	CO/N_2		
**1.**	Supported Flat sheet composite	Pebax 1657/ 40% [C_6mim][NTf_2]	163 Barrer	44	-	-	25 °C, variable pressure	This paper
**2.**	Neat polymer flat sheet	Pebax 1657	80 Barrer	70	-	-	21 °C, variable pressure	1
**3.**	Neat polymer flat sheet	Pebax 1657	20 GPU	28	50	0.5	25 °C, variable pressure	2
**4.**	Hollow fibers	Pebax 1657	7.6 GPU (69 Barrer)	18	13	1.1	25 °C, 2 bar	This paper
**5.**	Hollow fibers	Pebax 1657/ 40% [C_6mim][NTf_2]	23 GPU (245 Barrer)	8.7	12.3	0.7	25 °C, 2 bar	This paper
**6.**	Hollow fibers	Pebax 1657	86.4 Barrer	34	-	-	25 °C, 1 bar	3
**7.**	Hollow fibers	Pebax 1657/ 40% [emim][BF_4]	143 Barrer	34	-	-	35 °C, 3 bar	3
**8.**	Hollow fibers	[emim][NTf_2]	2600–3100 Barrer	33	-	-	25 °C, 1 bar	4
**9.**	Hollow fibers	Pebax 1657/ [emim][BF4]/GO	642 GPU	34	-	-	35 °C, 3 bar	5

(1) Yu, B.; Cong, H.; Li, Z.; Tang, J.; Zhao, X. S. Pebax-1657 Nanocomposite Membranes Incorporated with Nanoparticles/ Colloids/Carbon Nanotubes for CO2/N2 and CO 2/H2 Separation. *J. Appl. Polym. Sci.* **2013**, *130* (4), 2867–2876. https://doi.org/10.1002/app.39500.

(2) Park, C. H.; Lee, J. H.; Kim, N. U.; Kong, C. I.; Kim, J. H.; Kim, J. H. Solid-State Facilitated Transport of Carbon Monoxide through Mixed Matrix Membranes. *J. Memb. Sci.* **2019**, *592* (June), 117373. https://doi.org/10.1016/j.memsci.2019.117373.

(3) Fam, W.; Mansouri, J.; Li, H.; Chen, V. Improving CO2 Separation Performance of Thin Film Composite Hollow Fiber with Pebax®1657/Ionic Liquid Gel Membranes. *J. Memb. Sci.* **2017**, *537* (May), 54–68. https://doi.org/10.1016/j.memsci.2017.05.011.

(4) Kim, D. H.; Baek, I. H.; Hong, S. U.; Lee, H. K. Study on Immobilized Liquid Membrane Using Ionic Liquid and PVDF Hollow Fiber as a Support for CO2/N2 Separation. *J. Memb. Sci.* **2011**, *372* (1–2), 346–354. https://doi.org/10.1016/j.memsci.2011.02.025.

(5) Fam, W.; Mansouri, J.; Li, H.; Hou, J.; Chen, V. Gelled Graphene Oxide-Ionic Liquid Composite Membranes with Enriched Ionic Liquid Surfaces for Improved CO2 Separation. *ACS Appl. Mater. Interfaces* **2018**, *10* (8), 7389–7400. https://doi.org/10.1021/acsami.7b18988.
